# Effectiveness of Telemonitoring in Patients with Chronic Obstructive Pulmonary Disease in Taiwan-A Randomized Controlled Trial

**DOI:** 10.1038/srep23797

**Published:** 2016-03-31

**Authors:** Te-Wei Ho, Chun-Ta Huang, Herng-Chia Chiu, Sheng-Yuan Ruan, Yi-Ju Tsai, Chong-Jen Yu, Feipei Lai, The HINT Study Group, Yu-Feng Lin, Hung-Bin Tsai, Nin-Chieh Hsu, Chia-Lin Tseng, Chin-Chung Shu, Wen-Je Ko, Jin-Shing Chen

**Affiliations:** 1 Graduate Institute of Biomedical Electronics and Bioinformatics, National Taiwan University, Taipei, Taiwan; 2 Department of Internal Medicine, National Taiwan University Hospital, Taipei, Taiwan; 3 Department of Traumatology, National Taiwan University Hospital, Taipei, Taiwan; 4 Graduate Institute of Clinical Medicine, National Taiwan University, Taipei, Taiwan; 5 Department of Healthcare Administration and Medical Informatics, Kaohsiung Medical University, Kaohsiung, Taiwan; 6 Research Education and Epidemiology Center, Changhua Christian Hospital, Changhua, Taiwan; 7 School of Medicine, College of Medicine, Fu-Jen Catholic University, New Taipei, Taiwan; 8 Department of Computer Science and Information Engineering, National Taiwan University, Taipei, Taiwan; 9 Department of Electrical Engineering, National Taiwan University, Taipei, Taiwan; 10 Department of Surgery, National Taiwan University Hospital, Taipei, Taiwan

## Abstract

Chronic obstructive pulmonary disease (COPD) is the leading cause of death worldwide, and poses a substantial economic and social burden. Telemonitoring has been proposed as a solution to this growing problem, but its impact on patient outcome is equivocal. This randomized controlled trial aimed to investigate effectiveness of telemonitoring in improving COPD patient outcome. In total, 106 subjects were randomly assigned to the telemonitoring (n = 53) or usual care (n = 53) group. During the two months following discharge, telemonitoring group patients had to report their symptoms daily using an electronic diary. The primary outcome measure was time to first re-admission for COPD exacerbation within six months of discharge. During the follow-up period, time to first re-admission for COPD exacerbation was significantly increased in the telemonitoring group than in the usual care group (p = 0.026). Telemonitoring was also associated with a reduced number of all-cause re-admissions (0.23 vs. 0.68/patient; p = 0.002) and emergency room visits (0.36 vs. 0.91/patient; p = 0.006). In conclusion, telemonitoring intervention was associated with improved outcomes among COPD patients admitted for exacerbation in a country characterized by a small territory and high accessibility to medical services. The findings are encouraging and add further support to implementation of telemonitoring as part of COPD care.

Chronic obstructive pulmonary disease (COPD) is a leading cause of morbidity and mortality worldwide, and its prevalence and burden of COPD are projected to increase over the next decades^1^. In Taiwan, COPD ranks seventh among the common causes of death in 2010 and is estimated to cost approximately four billion New Taiwan dollars each year^2^. Despite advancements in pharmacologic therapy, patients with COPD often have debilitating symptoms that limit normal daily activities and impair quality of life^3^. Exacerbation of COPD, especially when hospitalization is required, is a major problem because of the negative effect on quality of life, prognosis, and medical costs^4^. There is an urgent need to reduce this burden, which has prompted the development of new COPD management strategies^5^.

Telehealth has shown promise in the management of chronic disease^6^,^7^,^8^. For patients with COPD, implementation of telehealth reduced re-admission, emergency room (ER) visits and disease exacerbation, and was shown to be cost-effective^9^. Telehealth, as a method of delivering healthcare to remote, resource-deprived areas, is not lacking in terms of evidence of benefit^10^; however, the value of its widespread use for monitoring purposes is much less clear. To date, most of the studies dealing with telemonitoring of patients with COPD have been performed in large countries^11^. Therefore, it should be investigated whether telemonitoring conveys similar advantages for patients with COPD in a small country.

The primary aim of the present study was to evaluate the effectiveness of a telemonitoring program to reduce COPD-related re-admission in Taiwan.

## Methods

### Study setting

This randomized controlled trial was conducted in the National Taiwan University Hospital, a tertiary-care referral center in Northern Taiwan, and participants were recruited between December 2011 and July 2013. Taiwan, an island of East Asia in the western Pacific Ocean with a total land area of about 36,000 square kilometers, established a universal National Health Insurance (NHI) program in 1995, and, by 2011, 99.9% of the 23 million individuals had been enrolled in the program^12^. This study was approved by the Research Ethics Committee of the National Taiwan University Hospital (201106097RB) and registered in the ClinicalTrials.gov (NCT01724684) in January 2012. Written informed consent was obtained from all study participants and all procedures were performed in accordance with relevant guidelines and regulations.

### Participants

All patients aged 20 years or older and admitted to the multidisciplinary combined care wards with a diagnosis of COPD were screened for eligibility. The wards accommodated patients primarily referred from the emergency service. Inclusion criteria included COPD exacerbation as the main diagnosis, current or former smokers, spirometry-confirmed airflow limitation (a value of forced expiratory volume in one second divided by forced vital capacity less than 0.7^1^), discharge to home, and accessibility to the internet and phone. Patients were excluded if they did not provide consent, were unable to access the study website, or had been enrolled in other trials.

### Study protocol

Patients were randomized to either the telemonitoring group or the usual care group using a computer-generated randomization scheme. Throughout the study, patients in both groups continued to receive usual care from their primary care physicians. For all study patients, a dedicated phone line was available for medical counseling provided by study nurses from 8 am to 8 pm on a daily basis.

A pulse oximeter, thermometer and sphygmomanometer were available for the telemonitoring group patients, and they were trained in the use of the equipment and an online diary by the study nurses prior to hospital discharge. The patients were instructed to report their symptoms using the electronic diary on the website each day for two months after discharge. The diary consisted of eight questions involving disease-related symptoms, vital signs and weight, and took about two min to complete. The submitted data were processed according to the predefined algorithm (Table 1), which was established by the study team in a round table conference. The indicators chosen and the scores assigned to each criterion were determined taking into account COPD symptomatology and common physiological responses to illness and by consensus. Once a warning was generated, the study nurses and attending pulmonologists received a notification to respond to the situation. The study pulmonologist would assess the patient’s data in light of the patient history, with the option of contacting and evaluating the patient by phone as clinically indicated. Based on the best clinical judgment, the patient could be referred to the clinic or ER.

### Data collection

Patient characteristics (age, gender, smoking status and pack-years of smoking, and body mass index), medical records (comorbidities, COPD medications and exacerbation history, and spirometry data) and outcomes were retrieved by a registered nurse blinded to the patient grouping. The comorbidities of interest included diabetes mellitus, hypertension, coronary artery disease and heart failure^1^. Major COPD medications were categorized as short-acting β_2_ agonists, long-acting β_2_ agonists, long-acting anticholinergics and inhaled corticosteroids. Data on COPD exacerbation were accessed in the previous year prior to enrollment of subjects, and hospitalization and ER visits due to exacerbation were documented.

### Outcome measures

The primary aim of the intervention was to reduce the frequency of re-admission. Accordingly, the primary outcome measure was the time to first hospital re-admission with a primary diagnosis of COPD exacerbation. Exacerbation was considered the primary diagnosis if the presenting symptoms were consistent with and the patients were treated for COPD exacerbation, and no other disease was managed as a priority. The secondary end points included (1) the time to first ER visit for COPD exacerbation, (2) the number of all-cause hospital re-admissions, and (3) the number of all-cause ER visits. All patients were followed up for six months after discharge and the endpoints were assessed at the end of the study period.

### Sample size estimation

It was assumed that the probability of re-admission was 50% for the usual care group at six months following hospital discharge, and the hazard ratio (HR) was 0.5 for the telemonitoring group^13^,^14^. To detect a statistically significant difference at the 0.05 level with a power of 0.8, it was calculated that a total of 116 patients should be included for randomization.

### Statistical analysis

Continuous variables were presented as means with standard deviations and categorical variables as frequencies with associated percentages. For intergroup comparisons, the independent sample t test and Fisher’s exact test were used for continuous and categorical variables, respectively. The Kaplan–Meier curves were plotted for time to first re-admission or ER visit due to COPD exacerbation, and the log-rank test was applied to test differences between two groups. Cox proportional hazard regression analysis was used to determine the effects of telemonitoring intervention on risks of re-admission and ER visit due to COPD exacerbation, as shown by an HR with 95% confidence interval (CI). All statistical analyses were two-sided and a p value of <0.05 was deemed statistically significant. The SPSS software (Version 15.0, SPSS Inc., Chicago, IL) was used for all data analyses.

## Results

### Patients

**Figure 1:**
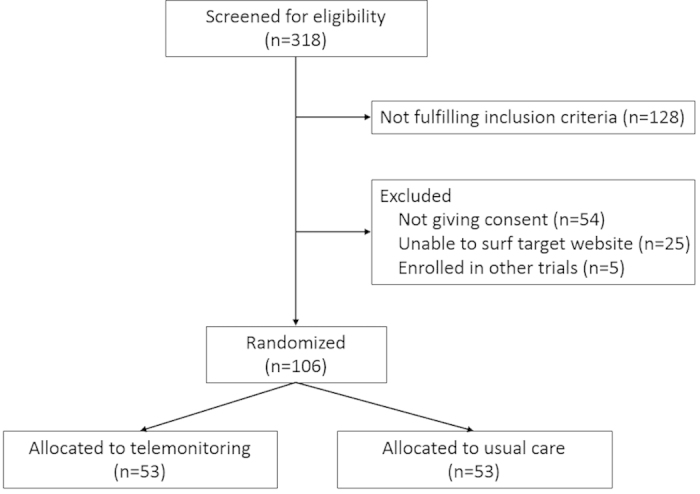
Study flow diagram.

During the study period, 318 hospitalized patients with a diagnosis of COPD were screened for eligibility (Fig. 1). A total of 106 patients were randomly assigned, 53 each to the telemonitoring and usual care groups. No participant withdrew consent during the course of the trial. The mean age of the study population was 80.2 ± 8.8 years at the time of enrollment and 76% of patients were men. About one-third of the patients were classified as having severe-to-very severe COPD according to the Global Initiative for Chronic Obstructive Lung Disease (GOLD) classification^1^. Demographics, pack-years of smoking, presence of comorbidities and disease severity markers were similar in both groups (Table 2).

### Time to first COPD-related re-admission and ER visit

**Figure 2:**
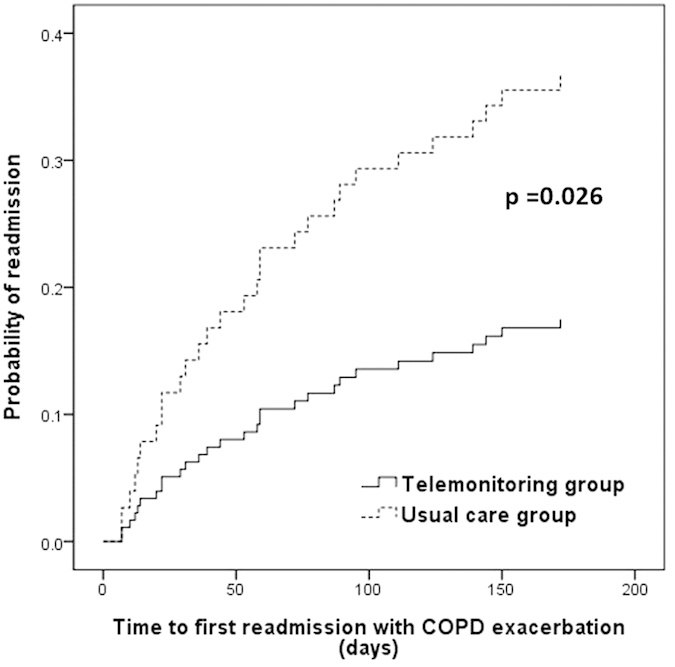
Kaplan-Meier curves showing the probability of readmission with COPD exacerbation. COPD, chronic obstructive pulmonary disease.

**Figure 3:**
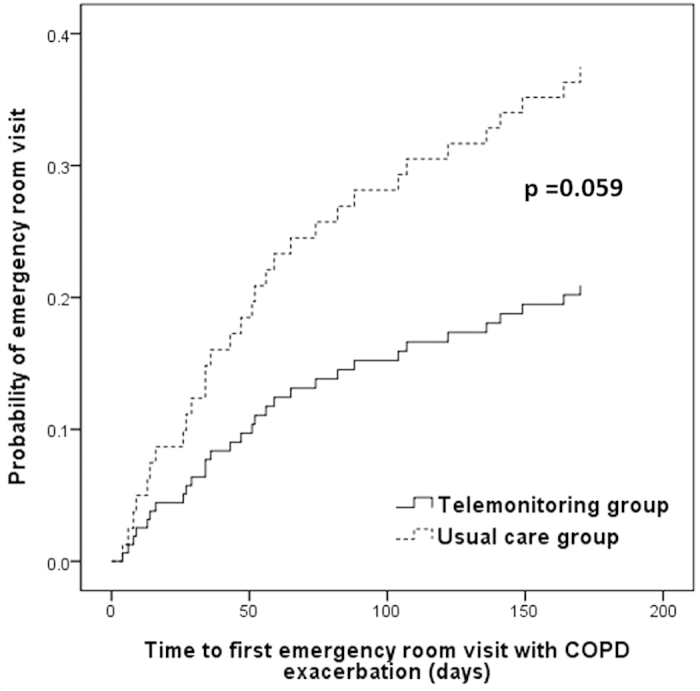
Kaplan-Meier curves showing the probability of emergency room visit with COPD exacerbation. COPD, chronic obstructive pulmonary disease.

As shown in Fig. 2, the time to first re-admission for COPD exacerbation was increased in the telemonitoring group as compared with the usual care group (p = 0.026 by log-rank test). At six months, the probability of COPD-related re-admission was significantly lower in the telemonitoring group (HR = 0.42; 95% CI = 0.19–0.92). In addition, telemonitoring intervention was associated with increased time to first COPD-related ER visit (Fig. 3), with an HR of 0.50 (95% CI = 0.24–1.04) over the six months of follow-up.

### Re-admission and ER visit

Telemonitoring intervention was associated with a significant reduction in the number of all-cause re-admissions from 0.68 to 0.23 per patient (p = 0.002) over a period of six months (Table 3). Similarly, patients in the telemonitoring intervention group had fewer ER visits for all causes than those in the usual care group (0.36 vs. 0.91 per patient; p = 0.006). Moreover, the telemonitoring group patients tended to have fewer episodes of COPD-related re-admissions (0.19 vs. 0.49; p = 0.11) or ER visits (0.23 vs. 0.55; p = 0.16) per capita than did usual care group patients.

### Medical counseling and responses to alerts

Twenty-one (40%) patients in the telemonitoring group made a total of 57 phone calls to the study team. Of those, 15 calls (five COPD related) were made to report new or altered symptoms. Ten patients (four COPD related) were referred to the ER; subsequently, four (two COPD related) of these were re-admitted. In the usual care group, 68 phone calls were made by 23 (43%) patients. Among these, 18 calls (five COPD related) were associated with new or altered symptoms, and 12 patients (four COPD related) required an ER visit. After initial assessment and management, seven patients (three COPD related) were later re-admitted. Between the two study groups, there were no significant differences in the number of patients making phone calls (21 vs. 23; p = 0.693) or the average number of calls per patient (1.1 vs. 1.3; p = 0.578).

A total of 192 alerts from 40 patients in the telemonitoring group were issued, 109 (57%) of which were judged to require a phone consultation from the study team. After the contacts, six alerts necessitated an ER referral and another six needed a referral to the clinic for further assessment. The remaining alerts were dealt with by health education, providing advice or guidance, observation, or reassurance. The remaining 83 (43%) alerts were considered innocent in that they were present while the patients were recovering from a worse situation.

Throughout the study period, there were no reports of serious adverse events related to the study procedures.

## Discussion

Among a set of patients discharged after hospitalization for COPD exacerbation, our results showed that telemonitoring intervention significantly postponed the time to first re-admission for exacerbation of COPD during a six-month follow-up. The telemonitoring group patients also, on average, had significantly fewer all-cause re-admissions or ER visits than the usual care group patients. In addition, a favorable effect of telemonitoring intervention on time to first ER visit for COPD exacerbation and on average number of re-admissions or ER visits due to exacerbation of COPD was observed. The main implication of this study was that in a country with a small territory and high accessibility to medical services, telemonitoring intervention remained associated with improved outcomes among patients discharged from hospital after COPD exacerbation.

Patients hospitalized for COPD exacerbation are at higher risk of re-admission in the following year^15^; thus, an important goal in patient care is to reduce these adverse events. A number of modalities, such as risk factor identification and intervention, self-management educational programs, and predischarge care bundles, have been utilized to achieve such a goal, but with diverse results^16^,^17^,^18^. The past decade has seen the growing use of telehealth as a possible approach to dealing with the increasing population with chronic diseases. In certain studies, telemonitoring has been shown to be beneficial in terms of exacerbation frequency, quality of life, ER visits, hospitalization and death among patients with COPD^19^,^20^. However, the diversity with regard to study populations, technology employed and components of the telehealth services has been high across the studies. Undoubtedly, there is an urgent need for further investigations to clarify the specific role of telemonitoring in the management of patients with COPD. The distinguishing features of the present study included the fact that it was conducted in a small, isolated island country, the study subjects were enrolled during admission for COPD exacerbation, and they were provided with easily accessible and affordable healthcare under the Taiwan NHI program. Therefore, our findings add to the existing knowledge by showing the effectiveness of telemonitoring intervention in terms of improving COPD patient outcomes in this specific setting.

Telehealth is a complex intervention and may include a variety of components, such as education, counseling, emotional support, remote monitoring and assisted planning^19^. Accordingly, when telehealth intervention is beneficial for the patients, it is difficult to pinpoint its active component. In this study, patients in both groups were provided with medical counseling via a phone call given that it was not only part of our routine practice but might help ease the patients’ anxiety and feeling of unsafety when they were randomly assigned to the usual care group. In this way, the only difference between the two study groups was the remote monitoring and response to changing signs and symptoms of patients with COPD. Early recognition and treatment of COPD exacerbations improves recovery, reduces risk of admission, and is associated with better quality of life^21^. Telemonitoring enables that early recognition and access to more timely treatment, thereby improving patient outcomes.

A key factor affecting the effectiveness of telemonitoring in COPD is the items monitored and corresponding algorithm. Prior studies have shown that there is a paucity of reliable early predictors of COPD exacerbation^22^. The parameters that we chose to monitor in this study were easily available and associated with COPD symptomatology, and the algorithm was determined somewhat empirically. Although no severe adverse events related to the study design were reported, a significant proportion of alerts were judged meaningless, with no action being required. Certainly, establishing ideal items for monitoring and algorithms with satisfactory sensitivity and specificity is a priority in the development of remote monitoring for COPD in telehealth. In this regard, combining advanced technologies for data processing and analysis with regularly updated clinical guidelines for COPD management is crucial^23^. Our design herein, at least a safe one, necessitates refinement in future work.

Although a recent review concludes that telemonitoring appears to have a positive effect in reducing COPD exacerbation and hospitalization, there are still few studies in this field and reported data are inconsistent in terms of methodology and conclusions^24^. Moreover, the largest trial to date demonstrated that telemonitoring had no significant clinical benefits but posed a substantial impact on workload for healthcare providers^25^. This trial, along with others using telemonitoring^26^,^27^, suggested that integration of this technology into existing best or comprehensive usual care does not improve COPD outcomes. Nonetheless, some may argue that the so-called best or comprehensive usual care is hardly a real-world practice and is probably not practical with respect to the large and growing COPD population. It remains to be determined what the best model of healthcare for COPD patients is in the coming studies^28^.

A number of limitations pertaining to this study should be mentioned. First, our sample size of this study did not allow for a subgroup analysis to define the most appropriate population for telemonitoring intervention, an important issue that is worth exploring in further studies. However, the sample size estimation was based on the primary outcome measure, and the predetermined significance level has been achieved in this study, except for the fact that the study included 106 patients as opposed to 116. Second, cost-effectiveness was not assessed in this study, because the study team was engaged in both study work and clinical service, and it was difficult to accurately calculate the direct and indirect personnel expense. In this regard, we are planning on an economic study to answer this question. Third, it was not possible to blind the study subjects and study personnel to treatment allocation given the interactive nature of the intervention. However, the outcome assessor was blinded to group allocation.

In summary, telemonitoring used to care for patients discharged for COPD exacerbation improves outcomes in terms of time to COPD-related re-admission, and average number of all-cause re-admissions and ER visits in the six-month follow-up. The findings are encouraging and promising, and add further support to the concept that telemonitoring is worth implementation as part of COPD care. Nevertheless, the favorable experience needs to be replicated in a large population under healthcare settings similar to those of Taiwan prior to widespread application of this modality.

## Additional Information

**How to cite this article**: Ho, T.-W. *et al.* Effectiveness of Telemonitoring in Patients with Chronic Obstructive Pulmonary Disease in Taiwan-A Randomized Controlled Trial. *Sci. Rep.* **6**, 23797; doi: 10.1038/srep23797 (2016).

## Figures and Tables

**Table 1:** Electronic diary scoring.

Item	Scoring
Weight	Score 1 if weight gain ≥1 kg in one dayScore 2 if weight gain ≥2 kg in three days
SpO_2_	Score 1 if <92%Score 2 if <90%
Temperature	Score 1 if ≥37.5 °CScore 2 if ≥38 °C
Heart rate	Score 1 if >100 beats/minScore 2 if >120 beats/min
Blood pressure	Score 1 if systolic pressure >160 or <100 mmHgScore 2 if systolic pressure >180 or <90 mmHg
Breathlessness	Score 1 if daily increase in mMRC Dyspnea Scale of 1 gradeScore 2 if daily increase in mMRC Dyspnea Scale of ≥2 grade
Sputum quantity	Score 1 if increase in frequency of expectoration of ≥50%
Sputum character	Score 1 if purulent
**Algorithm**	**If the sum of the scores is ≥2, an alert will be issued.**

SpO_2_, measurement of oxygen saturation via pulse oximeter; mMRC, Modified Medical Research Council.

**Table 2:** Baseline characteristics of the study population.

	Telemonitoring (n = 53)	Usual care (n = 53)	p value
Age, years	81.4 ± 7.8	79.0 ± 9.6	0.165
Male sex	43 (81)	38 (72)	0.253
Smoking, pack-years	58 ± 43	47 ± 31	0.143
Body mass index, kg/m^2^	20.2 ± 4.3	20.2 ± 4.1	0.930
Comorbidities			
Coronary artery disease	12 (23)	9 (17)	0.465
Heart failure	14 (26)	13 (25)	0.824
Hypertension	28 (53)	33 (62)	0.326
Diabetes mellitus	11 (21)	10 (19)	0.807
Exacerbation history in the previous year			
Admission	16 (30)	19 (36)	0.536
Emergency room visit	19 (36)	17 (32)	0.682
Spirometry			
FEV_1_ (%)	62 ± 23	62 ± 21	0.996
FEV_1_/FVC	0.53 ± 0.11	0.55 ± 0.09	0.314
GOLD classification of airflow limitation			
Mild/moderate	35 (66)	34 (64)	0.839
Severe/very severe	18 (34)	19 (36)	
Medications prior to admission			
Short-acting β_2_ agonist	47 (89)	45 (85)	0.566
Long-acting β_2_ agonist	32 (60)	35 (66)	0.546
Long-acting anticholinergic	36 (68)	34 (64)	0.682
Inhaled corticosteroid	33 (62)	37 (70)	0.412

Data were presented as mean ± standard deviation or number (%) as appropriate.

FEV_1_, forced expiratory volume in 1 second; FVC, forced vital capacity; GOLD, Global Initiative for Chronic Obstructive Lung Disease.

**Table 3:** Outcome measures at six months after discharge.

	Telemonitoring (n = 53)	Usual care (n = 53)	p value
Hospital readmission			
COPD exacerbation			
Total No. of episodes	10 (0–2)	26 (0–3)	
Episodes per patient	0.19 ± 0.44	0.49 ± 0.72	0.11
All causes			
Total No. of episodes	12 (0–2)	36 (0–3)	
Episodes per patient	0.23 ± 0.47	0.68 ± 0.94	0.002
Emergency room visit			
COPD exacerbation			
Total No. of episodes	12 (0–2)	29 (0–3)	
Episodes per patient	0.23 ± 0.47	0.55 ± 0.82	0.16
All causes			
Total No. of episodes	19 (0–2)	48 (0–7)	
Episodes per patient	0.36 ± 0.56	0.91 ± 1.29	0.006

Data were presented as mean ± standard deviation or number (range).

COPD, chronic obstructive pulmonary disease.
